# Engineered Human Muscle Tissue from Skeletal Muscle Derived Stem Cells and Induced Pluripotent Stem Cell Derived Cardiac Cells

**DOI:** 10.1155/2013/198762

**Published:** 2013-09-28

**Authors:** Jason Tchao, Jong Jin Kim, Bo Lin, Guy Salama, Cecilia W. Lo, Lei Yang, Kimimasa Tobita

**Affiliations:** 1 Department of Bioengineering, University of Pittsburgh, Pittsburgh, PA 15219, USA; 2 Department of Medicine, University of Pittsburgh, Pittsburgh, PA 15261, USA; 3 Department of Biomedical Engineering, Duke University, Durham, NC 27708, USA; 4 Department of Developmental Biology, University of Pittsburgh, Pittsburgh, PA 15224, USA; 5 McGowan Institute of Regenerative Medicine, University of Pittsburgh, Pittsburgh, PA 15219, USA; 6 Rangos Research Center, Room 8121, 4401 Pennsylvania Avenue, Pittsburgh, PA 15224, USA

## Abstract

During development, cardiac and skeletal muscle share major transcription factors and sarcomere proteins which were generally regarded as specific to either cardiac or skeletal muscle but not both in terminally differentiated adult cardiac or skeletal muscle. Here, we investigated whether artificial muscle constructed from human skeletal muscle derived stem cells (MDSCs) recapitulates developmental similarities between cardiac and skeletal muscle. We constructed 3-dimensional collagen-based engineered muscle tissue (EMT) using MDSCs (MDSC-EMT) and compared the biochemical and contractile properties with EMT using induced pluripotent stem (iPS) cell-derived cardiac cells (iPS-EMT). Both MDSC-EMT and iPS-EMT expressed cardiac specific troponins, fast skeletal muscle myosin heavy chain, and connexin-43 mimicking developing cardiac or skeletal muscle. At the transcriptional level, MDSC-EMT and iPS-EMT upregulated both cardiac and skeletal muscle-specific genes and expressed Nkx2.5 and Myo-D proteins. MDSC-EMT displayed intracellular calcium ion transients and responses to isoproterenol. Contractile force measurements of MDSC-EMT demonstrated functional properties of immature cardiac and skeletal muscle in both tissues. Results suggest that the EMT from MDSCs mimics developing cardiac and skeletal muscle and can serve as a useful *in vitro* functioning striated muscle model for investigation of stem cell differentiation and therapeutic options of MDSCs for cardiac repair.

## 1. Introduction

The adult heart is largely a nonregenerative organ. Although cardiomyocytes (CMs), the contractile cells of the heart, have a modest rate of turnover, ranging from 1% in youth to less than 0.5% in old age [1], this level is not enough to compensate for the large number of cardiomyocytes which are lost as a result of heart injury. Combined with the fact that heart disease is the leading cause of death in the United States [2], this has prompted the search for novel therapies to replace damaged myocardium. Muscle derived stem cells (MDSCs) and induced pluripotent (iPS) stem cells are among the types of stem cells under investigation for cardiac repair. MDSCs are a multipotent, somatic stem cell which can be obtained from skeletal muscle via a modified preplate method [3]. MDSCs can be rapidly expanded *in vitro* to obtain clinically relevant numbers of cells, which can be transplanted as an autologous graft. They are also advantageous because they are resistant to hypoxia, attenuate fibrosis, and readily differentiate into contractile cells [4]. We previously showed that rodent MDSCs differentiate into CM-like cells with cardiac-like electrophysiological, biochemical, and contractile properties using cell aggregate formation and 3-dimensional (3D) culture in a collagen-based scaffold [5], but engineered tissue models of human MDSCs in the context of their relationship to cardiac development and disease have not been investigated before. Studies have shown that cell aggregate culture can enhance cell-cell interactions and modulate gene expression, facilitating differentiation. Use of 3D engineered tissues as a vehicle for cell transplantation has been shown to provide a microenvironment which is optimal for cell survival and integration [6]. The iPS cells can be obtained from theoretically any somatic cell type by virus-mediated transfection of a quartet of reprogramming factors [7, 8]. These cells can then be differentiated into CMs or other cell types using established protocols [9, 10], which combine 3-dimensional culture with sequential growth factor and cytokine treatments. This approach ideally provides an unlimited source of CMs, but the modification of the genome of the host cell poses a challenge to clinical translation [11]. While cell therapy for heart disease remains a long-term goal in the field, our current aim is to provide a versatile and robust test bed to study striated muscle differentiation from stem cells towards this long-term goal.

Fetal gene expression is reactivated in the heart in response to various myocardial insults and disease states [12, 13], which includes expression of skeletal muscle specific proteins. However, this process remains poorly understood. The process may vary in different species, limiting the translatability of animal models, and conventional 2D *in vitro* models do not faithfully represent complex tissue architecture or allow for assessment of function at the tissue level. Direct biochemical and functional analyses on human myocardial tissue cannot take place due to limited tissue access *in vivo*. Therefore, it is necessary to develop better *in vitro* models of human cardiac muscle in order to better understand the relationship between striated muscle development (of both cardiac and skeletal muscle) and the pathogenesis of heart failure, which may lead to the development of better cell-based therapies. Creating better *in vitro* models to study human cardiac muscle development will not only broaden our understanding of developmental biology, it may enable us to develop better cell-based therapies for heart disease in the future.

Several studies have shown that developing cardiac and skeletal muscle shares expression of major cardiac or skeletal muscle specific contractile proteins and transcription factors [14–17]. However, this phenomenon has not been investigated in *ex vivo* engineered muscle tissues (EMT). Our current understanding of the nature of engineered muscle tissues is based on established models of mature cardiac and skeletal muscle. Thus, the objective of the current study was to investigate the occurrence of this “hybrid” phenotype in EMTs using two stem-cell-based models, human MDSCs and iPS-cell derived cardiac cells. Our hypothesis was that both MDSC-EMT and iPS-EMT possess properties of cardiac and skeletal muscle. Our results indicate that MDSC-EMT and iPS-EMT share a number of biochemical similarities, but iPS-EMT has a better degree of electrical coupling and adrenergic responsiveness.

## 2. Materials and Methods

### 2.1. Cell Culture

**Figure 1:**
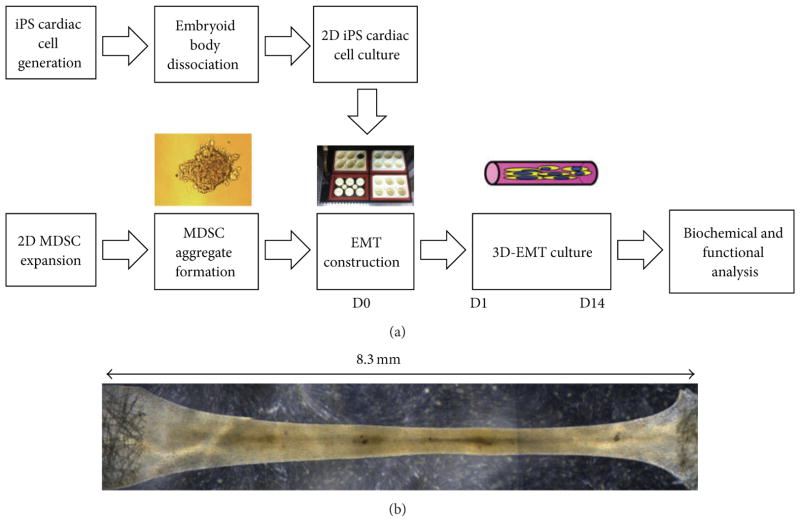
(a) Model system of 3D engineered muscle tissue culture. MDSCs undergo cellular aggregation prior to EMT construction. EMT is formed by mixing MDSC aggregates with collagen/ECM in a Flexcell Tissue Train Culture System. MDSC aggregates are cultured as EMT under low-serum conditions to induce differentiation. In an analogous manner, iPS-EMT is constructed by mixing dissociated iPS cardiac cells with collagen/ECM. (b) Composite phase contrast image of EMT. Cells in EMT aligned in the longitudinal direction and formed a muscle tissue during the 14-day culture period. Tissue morphology is maintained by anchors at each end of the tissue train plate. Individual sections of the EMT were imaged using a Leica light microscope at 2.5x magnification and merged using Adobe Photoshop.

MDSCs of 3 different human subjects (from 10 to 30 years old) were purchased from Cook Myosite, Inc. Cells were cultured in Cook Basal Media (Cook Myosite, Inc., Pittsburgh, PA, USA) supplemented with 10% Growth Supplement (Cook Myosite, Inc., Pittsburgh, PA, USA) and 1% Antibiotic-Antimycotic Solution (AAS, Invitrogen) on collagen-I coated flasks. Cells were used for no more than 4 passages and maintained below 50% confluence prior to initiation of differentiation. Human Y1 iPS cells were previously established in Lei Yang’s lab from healthy fibroblasts [10] and used as the control. iPS cells were maintained on mouse embryonic fibroblast (MEF) feeder layers with regular human embryonic stem cell medium containing 10 ng/mL FGF-2 [9]. iPS cells were differentiated into cardiomyocytes using our previously established protocol [9, 10]. All growth factors were from R&D systems. Following differentiation, embryoid bodies were dissociated using 0.05% trypsin (Invitrogen) and plated on Matrigel (BD Bioscience) coated 6 well plates in High-Glucose Dulbecco’s Modified Eagle Medium (DMEM, Cellgro) supplemented with 10% Fetal Bovine Serum (FBS), L-Glutamine (1 : 100, Invitrogen), and Penicillin Streptomycin (1 : 100, Invitrogen) (Figure 1(a)).

### 2.2. Fluorescence Activated Cell Sorting

Cryopreserved MDSCs from Cook Myosite were thawed and expanded to ~60% confluency. Cells were trypsinized and stained with CD45-APC (BD), CD56-PE-Cy7 (BD), CD146-PE (BD), and UEA-1-FITC (Ulex, BD) antibodies. Samples were analyzed on a Becton Dickinson (San Jose, CA) FACS Aria flow cytometer. The sorter is equipped with a standard configuration of three excitation sources. The FACS Aria contains blue, red, and violet lasers which excite at 488, 633, and 407 nm, respectively. Dead cells were initially eliminated based on 7-AAD, a dead cell exclusion dye. Cells were then analyzed on the basis of forward scatter (FSC-A) versus side scatter (SSC-A) for the selection of single cells and for the elimination of debris and aggregates of cells. This population was also analyzed on the basis of height versus width for both forward scatter and side scatter. Doublets of cells were eliminated on the basis of a high width measurement. Subsequent analysis of fluorescent populations was limited to this group. Unstained cells and isotype controls were used to set the background fluorescence. Single fluorochrome stained cells were used as compensation controls to eliminate spectral bleed over between the dyes. Cells were separated into CD56(+) and CD56(−) sub-populations. These were also determined to be CD45(−), eliminating hematopoietic subpopulations. The CD56(+)/CD45(−) and CD56(−)/CD45(−) were then analyzed for CD146 and Ulex expression. The FACSAria is equipped with an 85 *μ*m nozzle and the cells were sorted at a pressure of 35 psi. The larger nozzle size and lower pressure were used to increase the viability of the cells. Sterile PBS is run through the instrument as sheath fluid. Cells are collected into Cook Basal Medium with the populations as previously described [18–21].

### 2.3. Engineered Muscle Tissue (EMT) Construction

MDSCs were trypsinized, and MDSC-cell aggregates were obtained by rotation culture (Labnet Orbit 1000) on a suspension culture plate at 50 rpm for 24 hours prior to tissue construction [5]. iPS cardiac cells were trypsinized and counted on the day of EMT construction. Liquid rat tail collagen type I (3 mg/mL, Invitrogen) was neutralized with 0.1 N NaOH and mixed with Matrigel (BD Bioscience) with a collagen :Matrigel ratio of 0.8 and a final collagen concentration of 0.67 mg/mL. Cells were seeded in a collagen/Matrigel mixture at a density of 0.5 million cells per construct using a Flexcell Tissue Train Culture system (FX-4000, Flexcell International) with a total volume of approximately 200 *μ*L per construct to form a linear shaped construct (Figure 1(b)). To induce differentiation, MSDC-EMTs were cultured in Cook Basal Media supplemented with 5% growth supplement, 1% AAS, and human FGF-2 (5 ng/mL, Sigma). iPS-EMTs were cultured in DMEM supplemented with 10% Fetal Bovine Serum (FBS), L-Glutamine (1 : 100, Invitrogen), and Penicillin Streptomycin (1 : 100, Invitrogen).

### 2.4. Real-Time Polymerase Chain Reaction

Total RNA was prepared using Trizol solution (Invitrogen) and treated with TURBO DNA-free kit (Ambion, Austin, TX, USA). Primers, whose target genes are Nkx2.5, GATA4, Mef2A, Tbx5, GJA1, Myh6, Myh7, MyoD, myogenin, and beta-actin were obtained from Qiagen Quanti-Tect Primer Assay with the target fragment sizes approximately 100 base pairs. One step RT was performed using Applied Biosystems High Capacity RNA-to-cDNA Kit (Applied Biosystems) with a total of 2 *μ*g RNA in a total volume of 20 *μ*L with the following program: 37°C for 1 hour, 95°C for 5 minutes, 4°Chold. cDNA (1 *μ*L) was used for RT-PCR using the following program: 50°C for 2 minutes and 95°C for 10 minutes. This was followed by 95°C for 15 seconds and 60°C for 1 minute, repeated for 40 cycles. The final stage was 95°C for 15 seconds and 60°C for 15 seconds. SYBR Green was used as a detector. 3 samples were run for each target gene per group from pooled samples using Applied Biosystems 7900 HT system. Relative expression (RQ) was calculated using the *dd*Ct method with *β*-actin as an internal control. A Ct threshold of 38 was set based on the vendor’s instructions.

### 2.5. Immunohistochemical Staining

EMTs were fixed using 4% paraformaldehyde/PBS for 30 minutes and embedded in 13% polyacrylamide gel. 150 *μ*m thick sections were made using a vibratory microtome (Vibratome-1000, http://www.Vibratome.com) and stained for cardiac troponin-I (cTn-I, Abcam), cardiac troponin-T (cTn-T, Abcam), fast-skeletal muscle heavy chain (sk-fMHC, Sigma), connexin-43 (Cx-43, Abcam), CD31 (R&D Systems), Nkx2.5 (Santa Cruz), MyoD (Santa Cruz), and *α*-smooth muscle actin (*α*-SMA, Abcam) primary antibodies and Alexa Fluor 488 or Alexa Fluor 594 secondary antibodies. Stained samples were scanned using a confocal microscope (Olympus Fluoview FV1000) and used to generate 3D projection images of the tissue sections in ImageJ.

### 2.6. Mechanical Testing

The active force of EMTs was measured using a customized setup. Constructs were transferred to a muscle testing station perfused with warmed Ringer solution containing 2 mM CaCl_2_, 135 mM NaCl, 4 mM KCl, 10 mM Trizma-HCl, 8.3 mM Trizma-base, and 11 mM glucose. The constructs were attached to a force transducer (403A, Aurora Scientific, Aurora, Canada) using 10-0 mono-filament nylon sutures. The other end of each construct was attached to a micromanipulator. Field stimulation was applied using a stimulator (Grass S48 Stimulator, Grass Medical Instruments) at 5 ms duration, 100 V. The parameters were set at 10% above the threshold required to induce visible contraction of all constructs. The construct length was adjusted from 0% to 15% elongation. Force was measured at stimulation rates of 1–5 Hz and in response to isoproterenol (ISP, 1 *μ*M) and extracellular calcium (Calcium Chloride, 5 mM). Skeletal muscle stimulation was applied as follows to test if tetanus could be induced: 500 ms train rate, 1000 ms duration, and 20 Hz stimulation rate.

### 2.7. Intracellular Calcium Transient Measurement

Samples were loaded for 10 to 15 minutes at 37°C with Rhod 2-AM (Biotium) at a concentration of 5 *μ*g/mL. After the dye was loaded, the samples were placed on the heated stage of a Leica (DM LFSA) microscope. Optical mapping was performed with high spatiotemporal resolution (64 × 64 pixels, 176 fps) at 37°C using a Hamamatsu EM-CCD camera (Model C9100-12). Bipolar stimulation (20 V, 5 ms, 1–3 Hz, Grass S48 Stimulator, Grass Medical Instruments) was applied to electrically pace EMTs when necessary. The parameters were set at 10% above the threshold required to induce visible transients of all constructs. Calcium transients were recorded during spontaneous beating and during bipolar stimulation. Changes in signal were measured in response to isoproterenol (ISP, 1 *μ*M). Videos were processed in ImageJ (NIH) and analyzed using densitometry techniques. A3 × 3 pixel area was selected within each cell. The average pixel intensity within the region was calculated and normalized to the baseline resting value.

### 2.8. Statistical Procedures

One-way ANOVA was used to compare gene expression among experimental groups. A paired *t*-test was used to compare changes in force in response to isoproterenol and changes in force in response to extracellular calcium. An independent *t*-test was used to compare changes in calcium transient frequency in response to isoproterenol. Two-way repeated measures ANOVA was performed to compare active stress-strain relations. Data were expressed as mean ± standard error. Statistical significance was defined by a value of *P* < 0.05. All calculations were performed using Sigma Stat 3.0 software (Systat Software Inc.).

## 3. Results

### 3.1. Surface Marker Expression of MDSC

**Figure 2:**
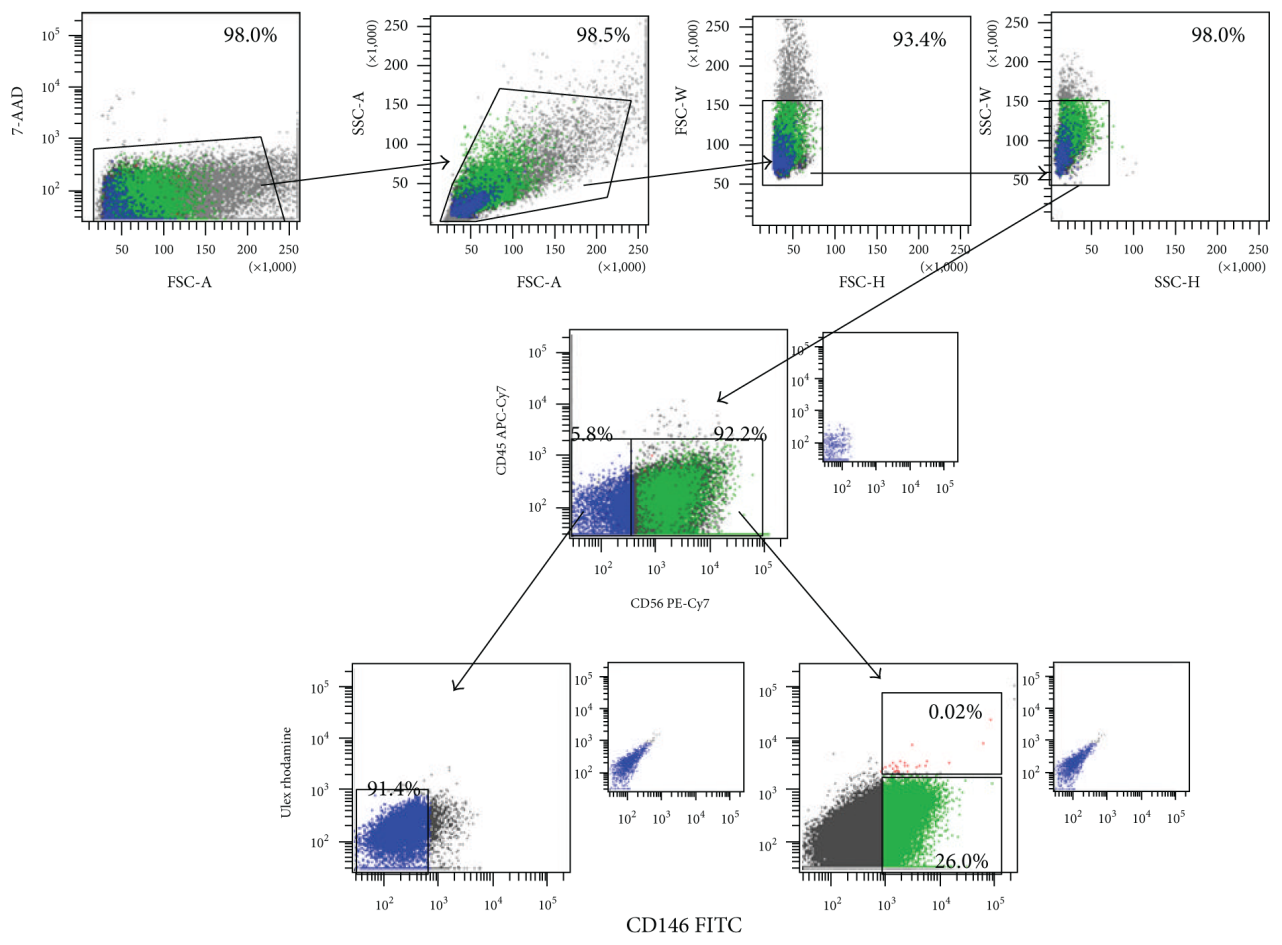
Fluorescence Activated Cell Sorting Analysis of undifferentiated human MDSCs. The majority of cells expressed myogenic (CD56) and mesenchymal (CD146) markers. A small number of cells expressed hematopoetic (CD45) and endothelial (Ulex) markers. Human MDSC population is composed of myoblasts (CD45−/CD56+/CD146−/Ulex−), myogenic-endothelial cells (CD45−/CD56+/CD146+/Ulex+), pericytes (CD45−/CD56−/CD146+/Ulex−), endothelial cells (CD45−/CD56−/CD146+/Ulex−), and myogenic-mesenchymal cells (CD45−/CD56+/CD146+/Ulex−).

We examined the surface marker expression profiles of undifferentiated MDSCs. MDSCs predominantly express CD56, a marker of myogenic lineage. A large number of cells also expressed CD146, a marker of mesenchymal stem cells. A smaller fraction of cells expressed endothelial cell marker Ulex (Figure 2, Table 1). The majority of cells were myogenic-mesenchymal cells, coexpressing CD56 and CD146 but not Ulex. Smaller fractions of cells were myoblasts (CD45−/CD56+/CD146−/Ulex−) and myogenic-endothelial cells (CD45−/CD56+/CD146+/Ulex+). Small populations of pericytes (CD45−/CD56−/CD146+/Ulex−) and endothelial cells (CD45−/CD56−/CD146−/Ulex+) were also present in sorted populations (Table 1). These data indicate that MDSCs are a heterogeneous population, which contains myogenic and supporting nonmyogenic cells.

### 3.2. Cardiac and Skeletal Muscle-Specific Gene and Protein Expression of EMT

In this study, a healthy Y1 human iPS cell line was differentiated into cardiac cells using our established method [10], which was modified from our previous cardiac differentiation protocol with human embryonic stem cells [9]. iPS cell derived cardiac cells and MDSCs were cultured in EMTs for 14 days.

**Figure 3:**
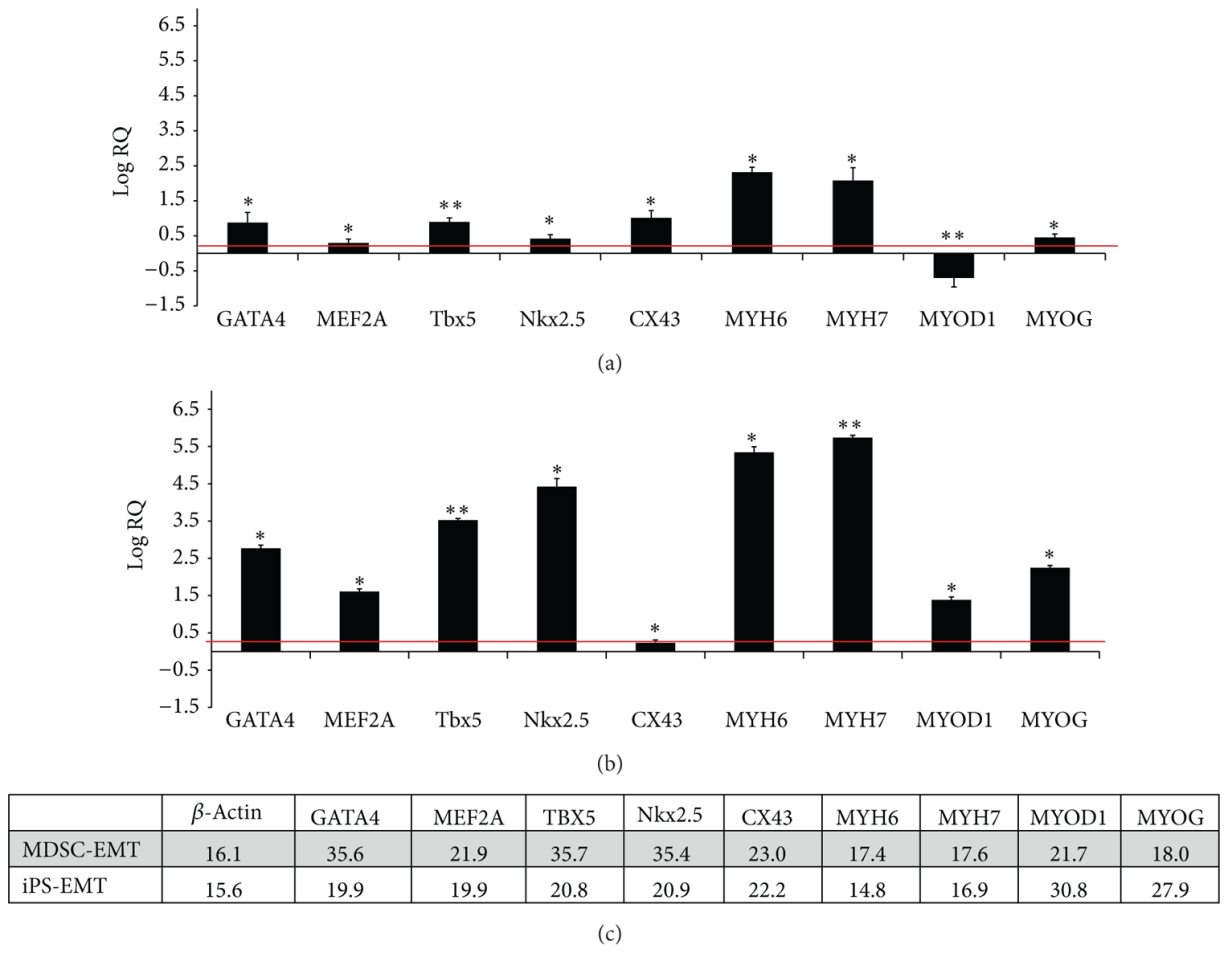
RT-PCR analysis of EMT Gene Expression. (a) Cardiac and skeletal muscle specific mRNA expression of MDSC-EMT at culture day 14 after tissue construction. Values are normalized to undifferentiated MDSCs. (b) Cardiac and skeletal muscle specific mRNA expression of culture day 14 iPS-EMT. Values are normalized to undifferentiated iPS cells. *P < 0.05. **P < 0.001. Red line indicates twofold increase. (c) Representative Ct values for MDSC-EMT and iPS-EMT. Although expression levels vary between the tissues, their transcriptional and structural gene profiles overlap.

We examined cardiac and skeletal muscle specific gene expression in MDSC-EMT and iPS-EMT at culture day 14 by quantitative RT-PCR. MDSC-EMT (Figure 3(a)) significantly upregulated expression of both cardiac (Nkx2.5, GATA4, MEF2A, Tbx5, Cx-43, Myh6, Myh7) and skeletal muscle (myogenin) genes compared to undifferentiated MDSCs. Note that relative quantification values are expressed as log_10_ scale. Interestingly, a major skeletal muscle transcription factor, MyoD, was downregulated (*n* = 9, *P* < 0.05). iPS-EMT (Figure 3(b)) significantly upregulated expression of both cardiac and skeletal muscle related genes compared to undifferentiated iPS cells (*n* = 6). While expression levels of certain genes vary between the two tissues, the key point is that they share a common gene expression profile at the transcriptional level, which opens up the possibility that MDSCs can transdifferentiate into cardiomyocyte-like cells under the appropriate conditions. On a similar note, the expression of skeletal muscle specific genes in cardiomyocytes derived from iPS cells using a widely used, established protocol suggests that the cardiac and skeletal muscle lineages are not mutually exclusive. These data show that MDSC-EMT and iPS-EMT share a common gene expression profile of cardiac and skeletal muscle transcription factor and structural protein genes similar to developing cardiac and skeletal muscle [14] and differentiating mesenchymal stem cells [22].

**Figure 4:**
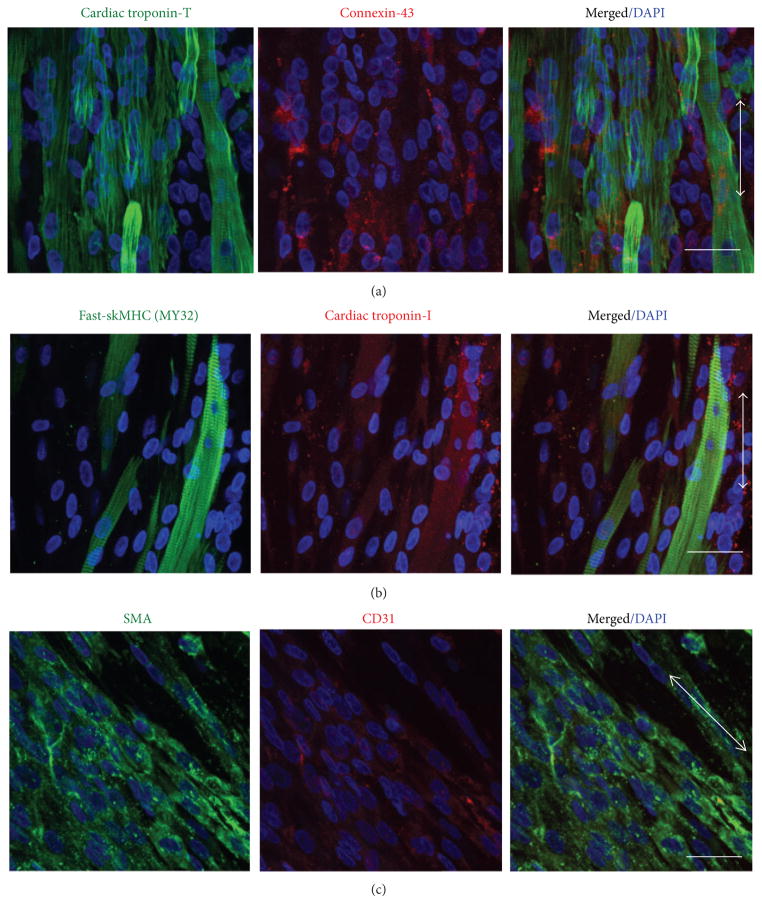
Immunohistochemical Analysis of culture day 14 MDSC-EMT. (a) Cardiac troponin-T (green) and gap junction protein Cx-43 (red). (b) sk-fMHC (green) and cardiac troponin-I (red). (c) *α*-Smooth muscle actin (green) and CD31 (red). Arrow indicates tissue orientation. Blue staining (DAPI) indicates nuclei. Scale bar indicates 30 *μ*m.

**Figure 5:**
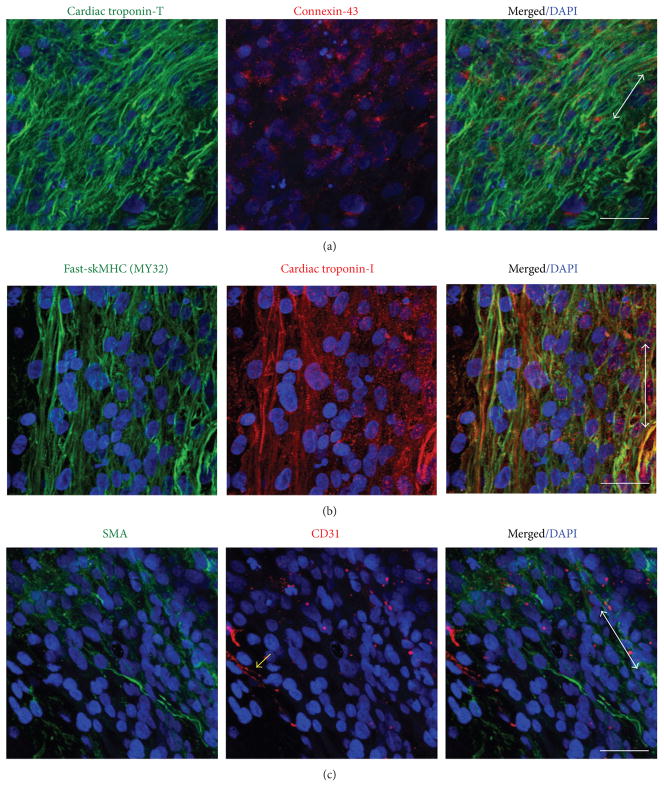
Immunohistochemical Analysis of culture day 14 iPS-EMT. (a) Cardiac troponin-T (green) and gap junction protein Cx-43 (red). (b) sk-fMHC (green) and cardiac troponin-I (red). (c) *α*-Smooth muscle actin (green) and CD31 (red). Yellow arrow shows representative CD31 staining. Arrow indicates tissue orientation. Blue staining (DAPI) indicates nuclei. Scale bar indicates 30 *μ*m.

Histologically, it is evident that MDSC-EMT and iPS-EMT coexpress cardiac and skeletal muscle structural proteins. Both EMTs express cTn-T in a striated pattern with gap junction protein, Cx-43 (Figures 4(a) and 5(a)). Notably, the expression pattern of Cx-43 in iPS-EMT resembles neonate myocardium. On the other hand, Cx-43 in MDSC-EMT appears to be more diffusely expressed and with less discrete localization at cellular junctions mimicking immature fetal myocardium [5]. In line with our previous findings, cTn-I and skeletal muscle specific sk-fMHC were found to be coexpressed (Figures 4(b) and 5(b)) similar to developing myocardium or skeletal muscle. Finally, nonmuscle proteins are also expressed within the tissue. We detected endothelial (CD31) and smooth muscle/mesenchymal cell (*α*-SMA) marker expression in iPS-EMT and MDSC-EMT (Figures 4(c) and 5(c)). The presence of CD31 and *α*-SMA together with the detection of a mixture of endothelial, mesenchymal, and myogenic surface markers by FACS supports the notion that EMT is complex, containing multiple cell types, which support cellular processes at the tissue level.

**Figure 6:**
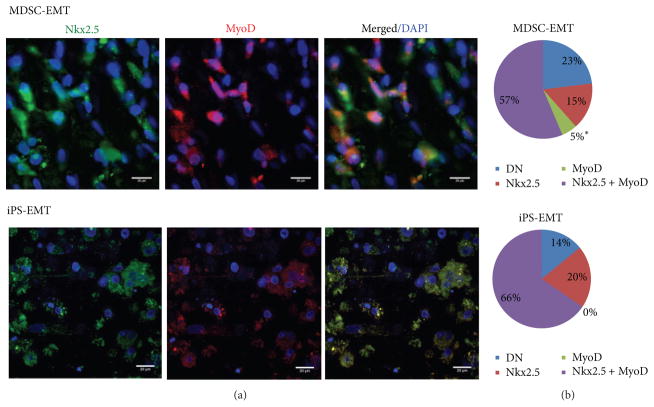
(a) Expression of Nkx2.5 and MyoD in MDSC-EMT and iPS-EMT. Both MDSC-EMT and iPS-EMT expressed Nkx2.5 (green) and MyoD (red) transcription factors at the protein level. Scale bar indicates 20 *μ*m. (b) Distribution of Nkx2.5 and MyoD positive cells in EMT. The majority of cells expressed both Nkx2.5 and MyoD. DN denotes double negative cells. * *P* < 0.05 by Chi-square test.

To investigate whether myocytes share cardiac and skeletal muscle-specific transcription factors at the protein level or exist as distinct populations of cells, we stained EMTs with Nkx2.5, a cardiac transcription factor, and MyoD, a skeletal muscle transcription factor (Figure 6(a), *n* = 3 fields each). In both MDSC-EMT and iPS-EMT, many cells expressed both Nkx2.5 and MyoD, suggesting that these cells have a true “hybrid” phenotype that does not simply arise from skeletal muscle contamination of cardiac cell cultures or vice versa. We did not observe any MyoD positive cells that did not express Nkx2.5 in iPS-EMT, demonstrating that MyoD expression is not the result of skeletal muscle contamination of iPS cultures. 15% of Nkx2.5 expressing cells in MDSC-EMT did not express MyoD, which may be related to the down-regulation of MyoD at the transcriptional level (Figure 6(b)). While it is tempting to speculate that these cells may be differentiating towards a more cardiac-like lineage, further studies are required to determine the final fate of these cells. A fraction of cells in MDSC-EMT expressed only MyoD, which was not observed in iPS-EMT. This fraction may contribute to some of the functional differences that were observed between MDSC and iPS-EMT.

### 3.3. Contractile Properties of MDSC-EMT and iPS-EMT

**Figure 7:**
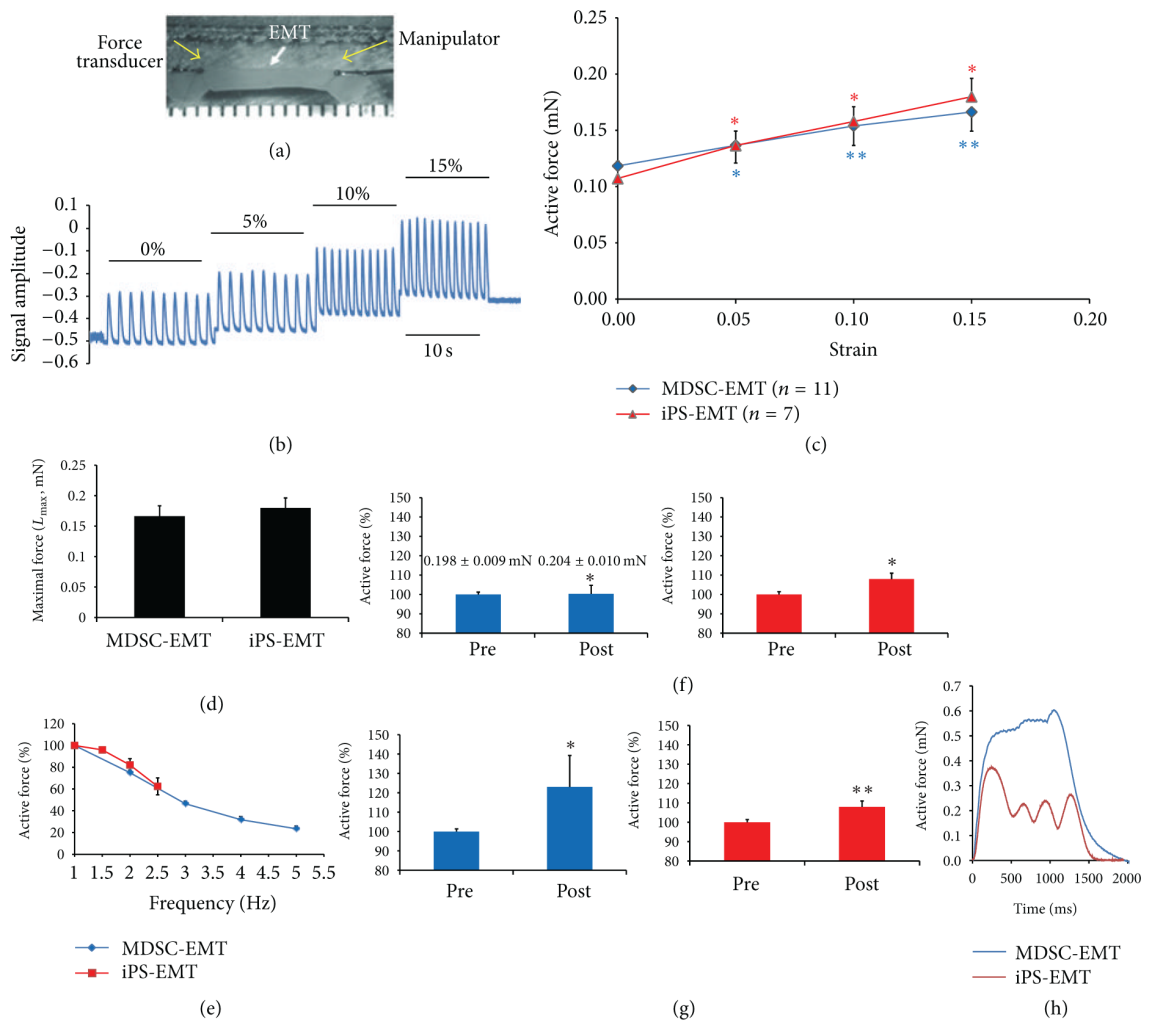
Biomechanical testing of EMT. (a) EMTs were mounted on a customized mechanical testing station using monofilament nylon sutures at the points indicated by the yellow arrows. (b) Representative contractile force traces of EMT at 0, 5, 10, and 15% strain. (c) Active force-strain relations of culture day 14MDSC-EMT and iPS-EMT at 0 to 0.15 strain. (d) Active force increased in response to increased strain (*P < 0.05, **P < 0.001 versus baseline, ANOVA). (d) Active force at maximal strain, *L*_max_(15%). (e) Force frequency relationship of EMT. Culture day 14 MDSC-EMT and iPS-EMT electrically field stimulated at rates of 1 to 5Hz at 100V and 5ms duration. EMTs showed decreased active force at increasing pacing rates. (f) Contractile response of EMT to ISP. Culture day 14 MDSC-EMT (blue) and iPS-EMT (red) were treated with 1 *μ*M ISP for 3 minutes and showed increased active force *P < 0.05 versus pretreatment. (g) Contractile response of EMT to extracellular calcium. Culture day 14 MDSC-EMT (blue) and iPS-EMT (red) were treated with 2mM CaCl_2_ for 3 minutes and showed increased active force. *P< 0.05 versus pretreatment. **P < 0.001 versus pretreatment. (h) Representative train stimulation of MDSC-EMT and iPS-EMT.

Both MDSC-EMT and iPS-EMT exhibited spontaneous beating activity. Single spontaneously beating cells were observed in MDSC-EMT as early as culture day 4, and spontaneous beating at the tissue level was observed by day 7 and sustained throughout the 14 day culture period. iPS-EMT exhibited spontaneous beating at day 3 which was also maintained for the duration of culture. EMT was attached to a force transducer in order to measure contractile force (Figure 7(a)). In response to field stimulation, both MDSC-EMT and iPS-EMT demonstrated the ability to generate contractile force. Both MDSC-EMT (*n* = 11) and iPS-EMT (*n* = 7) exhibited a Frank-Starling relationship, displaying a proportional increase in force with increasing construct length (Figures 7(b) and 7(c)). Some reports have stated that the force-strain relation is steeper in cardiac versus skeletal muscle [23], while others have found them to be similar [24]. Nonetheless, we have found that both share the same fundamental relationship with similar steepness. A greater steepness in cardiac muscle may manifest itself in mature tissues. iPS-EMT (0.179 ± 0.016 mN, *n* = 7) and MDSC-EMT (0.166 ± 0.017 mN, *n* = 11) exhibited similar contractile force at maximal length, *L*_max_ (Figure 7(d)). Both MDSC-EMT (*n* = 11) and iPS-EMT (*n* = 7) displayed negative force-frequency relationships in the range of 1 to 5 Hz stimulation rates (Figure 7(e)). iPS-EMT failed to respond above stimulation rates of 2.5 Hz, instead maintaining its own spontaneous cycling frequency. In response to nonselective *β*-adrenergic receptor agonist isoproterenol, iPS-EMT (107.91 ± 3.07%, *n* = 7, *P* < 0.05) and MDSC-EMT (101.44 ± 3.19%, *n* = 8, *P* < 0.05) showed a modest but significant increase in contractile force (Figure 7(f)). MDSC-EMT (123.05 ± 16.18%, *n* = 11, *P* < 0.05) and iPS-EMT (110.99 ± 5.76%, *n* = 7, *P* < 0.001) showed a significant increase in force in response to increased extracellular calcium ion (Figure 7(g)), suggesting that force generation is sensitive to extracellular calcium influx.

The negative force-frequency relationship is indicative of immature excitation-contraction coupling. Skeletal muscle can respond to adrenergic stimulation with increased inotropy [25], particularly under fatigued conditions [26]. However, under normal conditions, the response of skeletal muscle is small [27] but similar to early embryonic tissue [28]. On the other hand, the cardiac contractile response to ISP is much greater in later stages in cardiacmuscle [28]. From this, we can infer that immature cardiac and skeletal muscle both respond to ISP with modest positive inotropy. The extracellular calcium challenge is designed to test the acute response of the EMT to calcium influx. Addition of calcium leads to a transient increase in force [29] as calcium ions enter through cell membrane-bound calcium channels. Cardiac and skeletal muscle share isoforms of these calcium handling proteins during early myogenesis [30–32], and expression of certain proteins, particularly SERCA, is weak in early embryogenesis and increases during development. As a result, contraction is more dependent on extracellular calcium entry during the early phases of both cardiac and skeletal myogenesis [33–35]. Although we did not examine the expression of calcium handling proteins in this study, the acute extracellular calcium response suggests that the ability of myocytes within EMT to regulate cytosolic calcium is still immature and similar to embryonic or fetal myocytes [28]. While mature cardiac or skeletal muscle can also respond to extracellular calcium, the small inotropic response to ISP relative to the greater response to calcium demonstrates that beta-adrenergic receptor signaling and calcium cycling are not yet fully developed. Taken together with the negative force frequency relationship, these contractile responses to pharmacological and electrical stimulation indicate that EMT has contractile properties of immature striated muscle.

To further investigate the dependence of contraction on extracellular calcium, we treated EMTs with Cadmium Chloride (1 mM), a calcium channel blocker. After 5 minutes, contraction was significantly suppressed. The majority of MDSC-EMT and iPS-EMT constructs stopped contracting by visual inspection under a light microscope. (Data not shown.) This behavior does not occur in mature skeletal muscle [36]. Train stimulation at 20 Hz elicited a tetanic response from MDSC-EMT, while it did not in iPS-EMT (Figure 7(h)). In general, tetanus cannot be triggered in cardiomyocytes due to the unique electrical properties of myocardium, although it is possible under certain conditions [37, 38].

### 3.4. Intracellular Calcium Ion Transients

**Figure 8:**
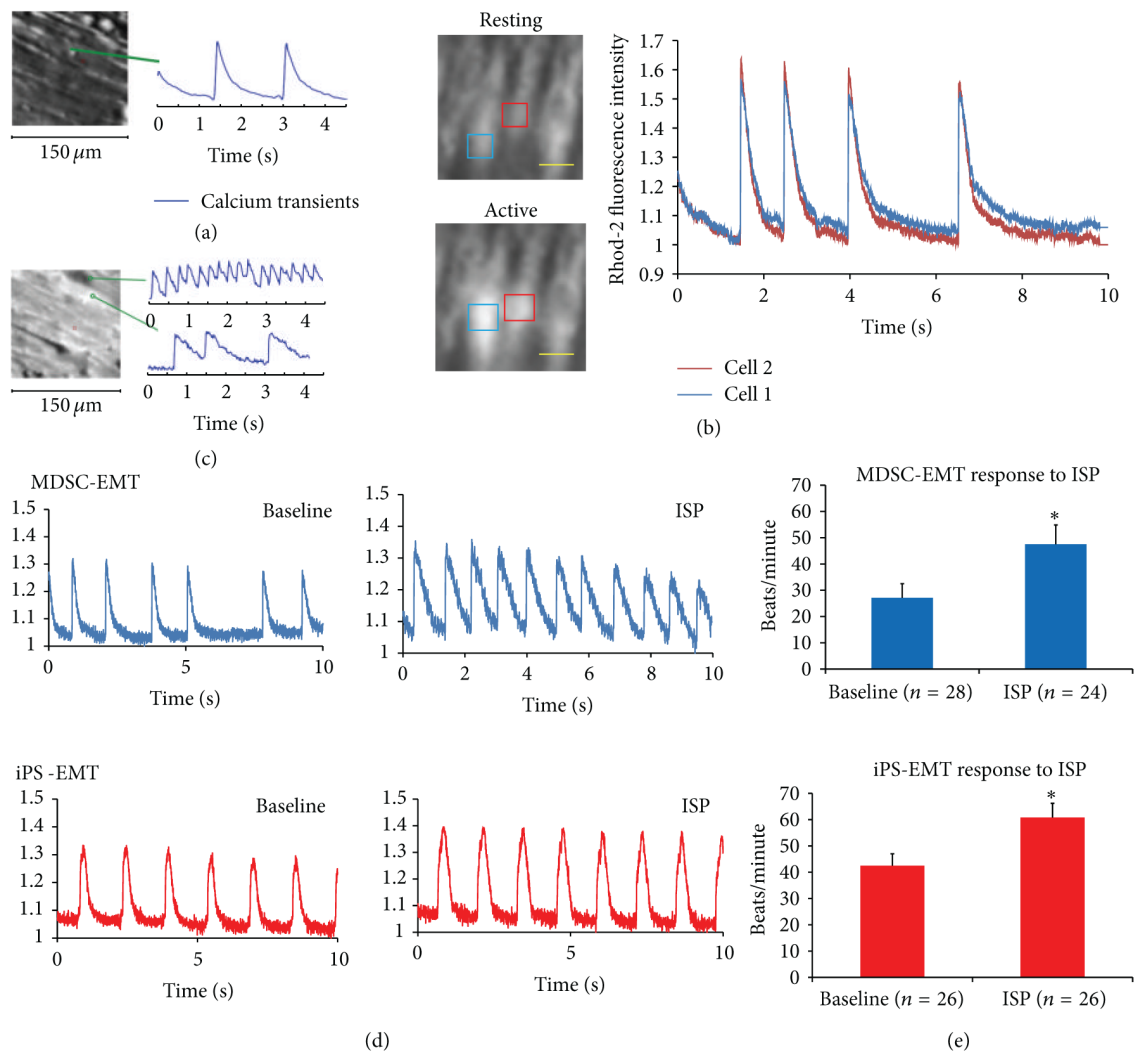
Intracellular Calcium Transients of EMT. (a) MDSC-EMT calcium transient under electric pacing at 1Hz, 20V, 5ms. (b) Synchronized calcium transients at rest (top) and during spontaneous beating (bottom) of cells within MDSC-EMT. (c) Unsynchronized spontaneous calcium transients in MDSC-EMT. Scale bar indicates 50 *μ*m. (d) Representative calcium transients of MDSC-EMT (blue) and iPS-EMT (red) at baseline and after addition of 1 *μ*M ISP. (e) Chronotropic effect of ISP on calcium transient activity of EMT. Treatment of culture day 14 MDSC-EMT and iPS-EMT with 1 *μ*M ISP increased spontaneous calcium transient activity. *P < 0.05 versus baseline.

To examine the electrophysiological properties of MDSC-EMT and iPS-EMT, we recorded intracellular calcium ion transients from culture day 14 EMTs. Transients were recorded using calcium indicator dye Rhod2-AM during spontaneous beating or under electrical pacing (1 Hz, 20 V, 5 ms). EMTs exhibited spontaneous calcium transients and responded to electrical pacing (Figure 8(a)). We noted that while iPS-EMT maintained steadier, rhythmic contractions (Figure 8(e)) compared to MDSC-EMT, MDSC-EMT contained a mixture of cells whose transients were synchronized (Figure 8(b), see Supplementary Video 1 in Supplementary Material available online at http://dx.doi.org/10.1155/2013/198762) and not fully synchronized (Figure 8(c)) with surrounding cells within the same engineered muscle tissue. These responses indicate immature calcium handling. Positive chronotropic effects of ISP on calcium transients of randomly selected cells within each EMT were observed in both EMTs (Figures 8(d) and 8(e)). We observed an increase in the frequency of calcium transients in both MDSC-EMT (*P* < 0.05) and iPS-EMT (*P* < 0.05).

## 4. Discussion

The widespread burden of cardiovascular disease has prompted the investigation of cell sources for cellular cardiomyoplasty. Among these, cells derived from skeletal muscle are an attractive source for cells. Unlike myocardium, which has limited regenerative ability, MDSCs have a robust potential for self-renewal as well as tolerance for hypoxic conditions and ability to reduce fibrosis and stimulate angiogenesis. In addition, unlike most other cell sources, they are naturally disposed to contractility. Although there are fundamental differences between mature cardiac and skeletal muscle (gap junction coupling, degree of multinucleation, morphology), there are multiple lines of evidence that support strong similarities between cardiac and skeletal muscle during development, which makes skeletal muscle a compelling source of stem cells. The Mef2 family of genes regulates many genes related to cardiac and skeletal muscle. Mef2c is first expressed in cardiac mesoderm and is used as an early marker of the cardiac lineage [39], but it is also expressed in MDSCs. Cardiac isoforms of Tn-T (cTn-T) are present in fetal skeletal muscle but are not expressed in healthy adult skeletal muscle [40]. Cardiac and skeletal actin are coexpressed in developing cardiac and skeletal muscle [41]. Skeletal muscle specific troponins are expressed in the developing heart [16, 42]. Cardiac-like excitation-coupling mechanisms are present in early skeletal myogenesis, while the “skeletal” excitation-coupling mechanisms dominate in more mature skeletal muscle [43]. Among the various cell types, which have been investigated for cardiac cell therapy, several studies have shown that MDSCs can readily transdifferentiate into a CM-like phenotype. There have been several previous reports of CM differentiation from rodent MDSCs, including those from our lab [5, 36, 44, 45]. To our knowledge, cardiomyocyte differentiation from human skeletal muscle derived stem cells has only been reported once [46]. Invernici and colleagues reported that retinoic acid induces cardiomyocyte differentiation in MDSCs. However, retinoic acid also enhances skeletal muscle differentiation in skeletal myoblasts [47], embryonic stem cells [48], and rhabdomyosarcoma cells [49]. While retinoic acid has been shown to accelerate CM differentiation [50], other studies have shown that it may even play an inhibitory role in cardiac specification [51]. Invernici et al. did not examine the potential for concurrent skeletal muscle differentiation. It is possible that skeletal muscle differentiation overlaps with cardiomyogenic differentiation, as we have reported here. An interesting study by Crippa et al. showed that MyoD expression in the heart is regulated by miR-669 [52], and ablation of this microRNA causes cardiac progenitor cells to differentiate into skeletal muscle. Taken together, these studies show that (1) developing cardiac and skeletal muscle share many similarities and (2) stem cells from skeletal muscle can differentiate into a cardiac-like phenotype and vice versa. However, the underlying mechanisms remain poorly understood. The current study was motivated by the need to develop a better model to study this phenomenon.

Recently, iPS cell derived cardiac cells have garnered interest as a source of cells to repopulate damaged myocardium. However, issues such as teratogenicity and viral-based genome modification limit clinical translation of this technology. We recently showed that developing cardiac and skeletal muscle share expression of cTn-I and sk-fMHC and major transcription factors [14]. To date, the question of whether engineered tissues created *in vitro* also exhibit this hybrid cardiac-skeletal phenotype has not been explored in detail. Gaining a better understanding of the nature of human EMT in the broader context of skeletal and cardiac muscle development, rather than exploring each one exclusively, may provide key insights towards advancing current cell therapies. In order to further probe this question, we studied EMT constructed using human iPS cell derived cardiac cells and human MDSCs.

Our strategy for differentiation of human MDSCs builds on our previous work with differentiation of rodent MDSCs [5]. The combination of low-serum conditions, cell aggregate formation, and 3D culture enhanced cardiac or skeletal muscle-specific gene expression and contractile properties in a way that better represents native cardiac or skeletal muscle tissue. This strategy, namely, using 3D culture, is analogous, though not identical, to the use of embryoid bodies (EB) to facilitate cardiac or skeletal muscle differentiation from pluripotent stem cells such as embryonic or iPS cells. It is worth noting that this strategy (EB formation) can be used to obtain both cardiac and skeletal muscle cells from pluripotent stem cells [53]. We found that both cardiac and skeletal muscle specific genes were upregulated during MDSC-EMT differentiation and iPS-EMT differentiation. This mirrors the coexpression of cardiac and skeletal muscle transcription factors that occurs during development of both muscle types, which become divergent in mature cardiac and skeletal muscle. The expression of skeletal muscle transcription factors in iPS cell derived CMs is interesting, since these transcription factors are generally considered to be skeletal muscle specific and not present in cardiac muscle [54]. The role that these transcription factors play in cardiac cells and their potential relation to the fetal gene program in heart failure remain to be investigated. We also found that most iPS cell-derived cardiomyocytes and MDSCs (>50%) coexpressed Nkx2.5 and MyoD protein, suggesting that these cells appear to be a hybrid phenotype of skeletal and cardiac muscle. The presence of MyoD in iPS-cell derived cardiomyocytes is interesting. There have been a few reports of MyoD expression in the hearts of adult, developing, or diseased organisms [55–57], but the prevailing consensus is that MyoD is specific to skeletal muscle [58, 59]. However, these reports are based on animal studies, and we are not aware of any reports of MyoD expression in the human fetal heart, either positive or negative. Thus, while there are numerous biochemical and functional similarities between engineered and native tissue, there may be more subtle differences between *in vitro* stem cell differentiation and developmental courses seen in native embryonic tissue. It is also possible that human heart development differs from other species in some respects. While fibroblasts can be programmed to cardiomyocyte-like cells by 3 factors (GMT) in mice, generation of cardiomyocytelike cells from human fibroblasts required a total of 7 factors to achieve a similar level of reprogramming [55]. This study highlights the as of yet poorly understood differences between cardiovascular development in humans and animals, which are important for translating basic research towards clinical therapies. Therefore, studies using human cells are important to complement the results of animal models.

While there were differences in expression of cardiac and skeletal muscle genes between iPS-EMT and MDSC-EMT, gene expression tells only one part of a much bigger story. Fibroblasts can be directly reprogrammed into cardiomyocytes by constitutive overexpression of 3 cardiac transcription factors (GATA4, Tbx5, andMef2c) [59]. Yet, only a small subset of these transduced cells develop into functioning cardiomyocytes despite high levels of expression of transduced genes. In this case, it is possible that more is not necessarily better. Generation of functional cells requires an overall balance of gene expression in the context of the source cell, desired target cell, and posttranscriptional factors. It has been suggested that ratios of selected genes rather than their absolute levels allow them to contribute to the development of functional cellular phenotypes [58]. In accordance with this study, the cardiac transcription factors Nkx2.5, GATA4, and Tbx5 are expressed in a ratio of approximately 1:1:1 in both iPS-EMT and MDSC-EMT. This balance may contribute to the cardiac-like phenotypes that were observed in both EMTs.

These trends also manifest themselves histologically. We found that cTn-I and sk-fMHC are coexpressed by both MDSC-EMT and iPS-EMT, similar to developing cardiac and skeletal muscle [14]. Both EMT types also express cTn-T and Cx-43. A noteworthy difference is that the expression of Cx-43 in MDSC-EMTs at the cell-cell interface as discrete gap junctions is less pronounced compared to iPS-EMT. However, we did observe synchronous calcium transients between neighboring myocytes in MDSC-EMT, suggesting the potential for electrical coupling. Gap junction coupling is an important requirement for synchronized contraction between myocytes, and lack of electromechanical integration was identified as a major hurdle for the use of skeletal myoblasts for myocardial repair [60]. While cells in MDSC-EMT did not form gap junctions to the same extent as iPS-EMT during the culture duration in the current study, connexin- 43 gap junction formation is upregulated prior to myoblast alignment, and overexpression of connexin-43 in myoblasts [61–63] and mechanical preconditioning [64, 65] have been shown to enhance electrical coupling with cardiomyocytes. Thus, strategies to improve cell-cell coupling in skeletal muscle cells are ongoing.

Both MDSC-EMT and iPS-EMT demonstrated a chronotropic response to ISP similar to cardiac tissue and distinct from mature skeletal muscle [36]. Both MDSC-EMT and iPS-EMT exhibit positive force-strain (Frank-Starling) and negative force-frequency relationships, which are indicative of immature excitation-contraction coupling. Taken together, our functional assessments show that MDSC-EMT and iPS-EMT mimic immature or developing striated muscle.

A number of studies have shown that a mixture of cell types is optimal for efficient CM differentiation and tissue formation [66]. Our flow cytometry data indicates that MDSC is a predominantly myogenic-mesenchymal-like population with smaller numbers of skeletal myoblasts, myogenic-endothelial cells, pericytes, and endothelial cells [19]. We also show expression of smooth muscle (*α*-SMA) and CD31 in the construct. iPS-EMT also contains a mixture of cardiomyocytes, smooth muscle, and endothelial cells [10]. Thus, both tissue types contain a naturally heterogeneous mixture of cells which supports efficient muscle differentiation and tissue formation. The role that these heterotypic cell interactions play in striated muscle development and growth requires further investigation.

There are a number of limitations to the current study. Our study compared the properties of two types of artificial human striated muscle and showed that both share cardiac and skeletal muscle proteins/genes and functional properties. We did not have access to native human tissues for this study, so it remains to be seen if these properties are also present in either developing or mature human tissues. However, our findings are consistent with previous *in vitro* and animal studies, and we believe these findings provide new insight into previously unknown properties of engineered tissues. Given our previous findings, further studies are needed to better understand the role of the “hybrid” phenotype in cell fate decisions. Our study also calls into question the specificity of commonly used cardiac and skeletal muscle markers. When evaluating cell differentiation, the possibility of differentiation into other, closely related cell types should also be considered. This also adds complexity to evaluating cardiac and skeletal muscle differentiation. Differentiated myocytes likely express a combination of cardiac and skeletal muscle specific proteins. Posttranscriptional and epigenetic factors may be an important key in generating more functional CM-like cells. Finally, we identified both muscle and nonmuscle cell types within each EMT as part of a heterogeneous population. Whether differentiated muscle cells arise from a single subpopulation or multiple subpopulations is unclear, and the role of these cell interactions in cell differentiation and tissue formation requires further investigation.

## 5. Conclusion

In summary, we have shown that MDSC-EMT and iPS-EMT share major cardiac and skeletal muscle specific gene and structural protein expression patterns and highlighted their functional similarities/differences. These 3D muscle tissue models recapitulate elements of developing cardiac and skeletal muscle. While the optimization of cell sources for cardiomyoplasty remains an area of ongoing work in the field, our results show that EMT mimics developing cardiac and skeletal muscle and can serve as a versatile *in vitro* model to study and better understand cell differentiation and tissue development as a functional human engineered muscle towards the long-term goal of cellular cardiomyoplasty.

## Figures and Tables

**Table 1:** FACS analysis of MDSC.

	Positive %
CD45	1.1 ± 1.0 (*n* = 3)
CD56	93.1 ± 6.2 (*n* = 5)
CD146	84.3 ± 5.9 (*n* = 5)
Ulex	1.9 ± 1.8 (*n* = 5)
Cell type	
CD45−/CD56+/CD146−/Ulex−	2.87 ± 0.68
CD45−/CD56+/CD146+/Ulex+	1.79 ± 0.78
CD45−/CD56−/CD146+/Ulex−	1.55 ± 0.98
CD45−/CD56−/CD146−/Ulex+	0.02 ± 0.01
CD45−/CD56+/CD146+/Ulex−	81.26 ± 3.12

## Abbreviations

CM: Cardiomyocyte
MDSC: Muscle derived stem cell
EMT: Engineered muscle tissue
iPS cell: Induced pluripotent stem cell
ISP: Isoproterenol
Cx-43: Connexin-43
cTn-I: Cardiac specific troponin I
cTn-T: Cardiac specific troponin T
Sk-fMHC: Fast skeletal myosin heavy chain
*α*-SMA: Alpha smooth muscle actin
